# Natural acetylation impacts carbohydrate recovery during deconstruction of *Populus trichocarpa* wood

**DOI:** 10.1186/s13068-017-0734-z

**Published:** 2017-02-23

**Authors:** Amanda M. Johnson, Hoon Kim, John Ralph, Shawn D. Mansfield

**Affiliations:** 1 Department of Wood Science, Faculty of Forestry, University of British Columbia, Vancouver, BC Canada; 2 Department of Biochemistry, University of Wisconsin, Madison, WI USA; 3 Department of Energy Great Lakes Bioenergy Research Center, Wisconsin Energy Institute, Madison, WI USA

**Keywords:** Acetate, Acetyl group, Xylan, Pretreatment, Biorefinery, Advanced biofuels

## Abstract

**Background:**

Significant variation in the inherent degree of acetylation naturally exists in the xylem cell walls of *Populus trichocarpa*. During pretreatment, endogenous acetate hydrolyzes to acetic acid that can subsequently catalyze the breakdown of poplar wood, increasing the efficiency of biomass pretreatment.

**Results:**

Poplar genotypes varying in cell wall composition were pretreated in 0.3% H_2_SO_4_ in non-isothermal batch reactors. Acetic acid released from the wood was positively related to sugar release during pretreatment (*R* ≥ 0.9), and inversely proportional to the lignin content of the poplar wood (*R* = 0.6).

**Conclusion:**

There is significant variation in wood chemistry among *P. trichocarpa* genotypes. This study elucidated patterns of cell wall deconstruction and clearly links carbohydrate solubilization to acetate release. Tailoring biomass feedstocks for acetate release could enhance pretreatment efficiencies.

**Electronic supplementary material:**

The online version of this article (doi:10.1186/s13068-017-0734-z) contains supplementary material, which is available to authorized users.

## Background

In recent decades, there has been widespread global interest in developing dedicated bioenergy feedstocks [1]. Sustainable and economically viable production of purpose-grown lignocellulosic feedstocks is requisite on the ability to generate crops with suitable composition and significant biomass yield to maximize land-use efficiency. Biochemical conversion of lignocellulosic biomass can yield valuable bioproducts, including ethanol, organic acids, lignin-derived coproducts, butanol, and hydrogen gas [2]. Tailoring lignocellulosic feedstocks for emerging biorefineries, through classical breeding or biotechnological application, could offer a means to improve the economy and feasibility of commoditizing these bioproducts [3]. Poplar wood has emerged as a promising biofuel feedstock, as these trees have large native ranges, inherently possess the ability to grow on marginal sites, and are highly productive.

Cell wall ultrastructure and composition dictate the utility of poplar and other lignocellulosic feedstocks for bioconversion applications. Lignocellulosic feedstocks are largely recalcitrant to breakdown into utilizable sugars because of the compact structure of crystalline cellulose microfibrils, lack of substrate porosity, and high lignin concentrations [4]. Consequently, harsh pretreatment regimes are often required to separate the carbohydrates from lignin in the cell wall complex and provide sufficient accessibility for biochemical conversion. Some pretreatments, notably those using dilute acid processes, solubilize hemicelluloses into the reaction liquor, where they degrade or are discarded as a waste stream [5, 6]. As such, the hemicelluloses, comprising up to 30% of the secondary cell wall of poplar [7], are an underutilized fraction of biomass. Studies of their dissolution and degradation are therefore required in order to improve estimates of product yield in the biorefinery process.

Hemicelluloses are highly branched mixed-carbohydrate polysaccharides with a degree of polymerization of approximately 200 [8]. Xylan, which has a backbone of β-(1→4)-linked xylosyl residues, is the main hemicellulose in poplar. Acetate groups decorate xylopyranosyl residues, such that acetate makes up approximately 5% (w/w) of poplar wood [9].

To date, the biological and structural roles of acetate substitution are uncertain. Acetylation may prevent aggregation of xylan precursors during their biosynthesis and transport to the cell wall because of its associated steric forces [10]. In the assembled secondary cell wall, xylan acetylation has been shown to increase chain stiffness and impact the flexural properties of wood, including modulus of rupture and modulus of elasticity [11, 12]. Recent research is uncovering the regularity of acetate substitution, and suggests that acetate groups face towards the lignified region of the cell wall to improve cohesion between lamellae [13, 14].

Acetate has been the subject of research because of its effect on wood processing, pulping, and bioconversion. Acetate corrodes metal, decreases fiber swelling, and inhibits growth of fermentative microorganisms [15–17]. Studies also suggest that acetate from lignocellulose impacts the pretreatment phase of bioconversion operations [18]. In most lignocellulosic substrate pretreatment regimes, a key goal is to remove hemicelluloses from the cell wall matrix and offer a means to liberate and extract the recalcitrant lignin polymer [19, 20]. Although acetate is removed during alkaline pretreatment, it remains part of biomass during most other types of pretreatment [21]. Currently, dilute acid pretreatment, which hydrolyzes glycans and disrupts hydrogen bonding between cell wall polymers, is the most commonly considered pretreatment. Xylan deconstruction usually occurs in three phases during dilute acid pretreatment. In the first phase, fast- and slow-reacting xylan are dissolved and hydrolyzed to oligomers [22]. Next, oligomers are further hydrolyzed to individual xylose monomers, and acetate hydrolyzes to acetic acid [23, 24]. During the third phase, xylose monomers dehydrate to furfural, while hexose monomers degrade to 5-hydroxymethyl-2-furaldehyde (HMF) [25].

Acetic acid derived from acetate in wood has both positive and negative effects on biomass conversion. Acetic acid alone can be an effective agent for selective delignification [26]. The powerful dissolving effect of acetic acid led to the establishment of acetosolv pretreatment for hardwood, softwood, and agricultural residues [27–31]. For example, pretreatment of beechwood with 1% acetic acid was shown to be as effective as raising the reaction temperature by 20 °C [32].

In contrast, during biochemical pretreatment regimes that employ enzymatic digestion, acetate increases the overall enzyme load required to effectively convert woody feedstocks [18]. Acetate accumulating above 100 mM in pretreatment slurries was shown to be inhibitory to downstream fermentative microorganisms [33]. These negative effects have prompted several studies focused on acetate removal from pretreated biomass prior to enzymatic conversion [34–36]. In one study, transgenic *Arabidopsis* stems with 32% less total cell wall acetate content yielded 70% higher ethanol production by fermentation compared to wild-type stems [37]. This study highlights the importance of acetate content in lignocellulosic biorefinery processes, as acetate has been shown to be both positively or negatively correlated with sugar release in previous studies depending on the pretreatment and hydrolytic method employed. Herein, the dissolution of xylan, glucan, and acetate groups during pretreatment of poplar wood are explored.

## Results

### Wood sampling and degree of acetylation

Wood sampled from 200 unrelated 5-year-old *Populus trichocarpa* individuals grown in a common garden had an average acetate content of 5.2 ± 0.3% (w/w ± SD, extractives-free dry weight), with a high of 6.7% and low of 3.5% w/w. Regression analysis of several wood chemistry traits of the trees determined whether acetate content correlates with any of the primary chemical features of the wood (Table 1; Additional file 1: Table S1). There were positive correlations between xylose, mannose, and rhamnose and acetate content (*R* = 0.40, 0.28, and 0.25, respectively), whereas glucose and galactose contents were inversely correlated with acetate content (*R* = −0.40, −0.16). Acid-soluble lignin and acetate were positively correlated (*R* = 0.35; Table 1). The Klason lignin (acid-insoluble lignin) or arabinose content of the wood samples were not significantly associated with acetate (*R* = 0.07, 0.02), nor were the 5-year growth traits, including total tree biomass (*R* = 0.03).

**Table 1:** Associations between wood acetate content and other cell wall components or traits among *P. trichocarpa* genotypes

	*R*	*p* value	*n*
Glucose	−0.40	<0.001	208
Xylose	0.40	<0.001	203
Lignin (acid-soluble)	0.35	<0.001	207
Mannose	0.28	<0.001	202
Rhamnose	0.25	<0.001	205
Galactose	−0.16	0.004	204
Lignin (Klason)	0.07	0.20	208
Total tree biomass	0.03	0.67	232
Arabinose	0.02	0.78	208

*R* Pearson correlation coefficient; *p* value, test statistic; *n* number of observations. Values are the average of three technical replicates. Raw data are presented in Additional file 1: Table S1

### NMR

**Fig. 1:**
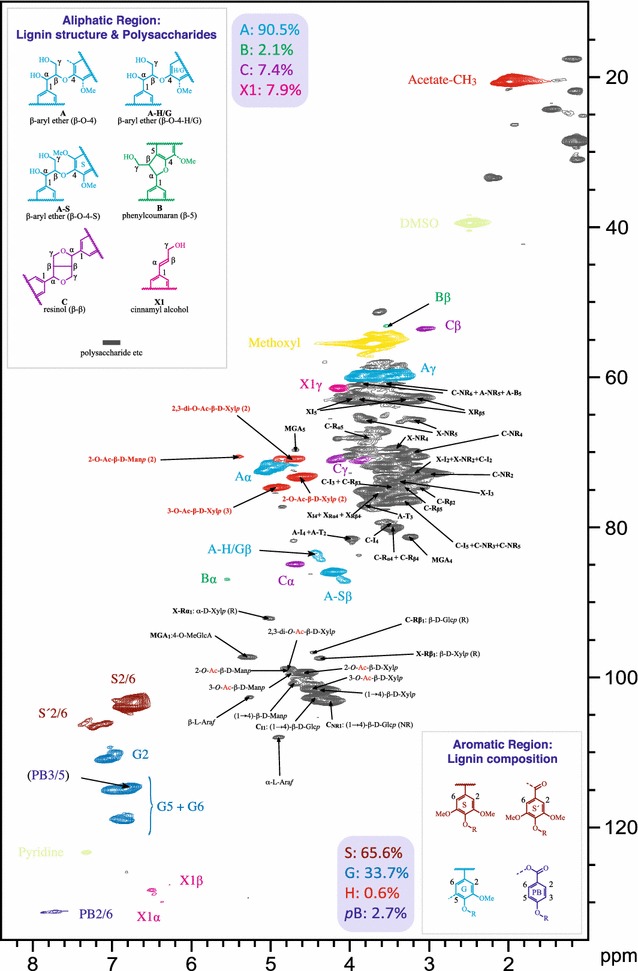
HSQC 2D-NMR spectrum obtained on total cell wall material from poplar wood

Figure 1 is a 2D ^1^H–^13^C-correlated (HSQC) NMR spectrum of poplar xylem. Acetate groups are positioned on xylopyranosyl and mannopyranosyl residues. Considerable amounts of xylopyranosyl residues are *O*-acetylated, whereas the majority of xylan structures are non-acetylated. The peaks of 2-*O*-Ac-β-d-Xyl*p* C2/H2 at 73.5/4.64 ppm and a 3-*O*-Ac-β-d-Xyl*p* C3/H3 at 75.0/4.94 ppm can be easily recognized. Poplar inherently displays moderate levels of 2,3-di-*O*-acetylation of xylopyranosyl units, a feature that gives a discernable correlation (C2/H2) at 71.0/4.74 ppm. The 2-*O*-Ac-β-d-Man*p* C2/H2 contour is smaller than the 3-*O*-Ac-β-d-Xyl*p* C3/H3 contour, which is marginally smaller than the 2-*O*-Ac-β-d-Xyl*p* C2/H2 contour. This suggests a relative abundance of 2-*O*-acetylated mannopyranosyl < 3-*O*-acetylated xylopyranosyl < 2-*O*-acetylated xylopyranosyl units in poplar cell walls. MeGlcA (4-*O*-methyl-α-d-glucuronic acid) and α-l-arabinofuranosyl (α-l-Ara*f*) residue units were also detected. Acetate groups on poplar lignin are not apparent, in part because of spectral congestion in the Cγ/Hγ region, and most acetate groups are on the hemicellulosic components. Although we have convincing, repeatable evidence that the acetate values in high-, medium-, and low-acetate lines are approximately 7, 5, and 3%, extensive investigation by whole-CW NMR does not allow us to delineate from where the changes originate. As seen from the spectrum and the data, only minor differences in the acetate levels in total or on the xylopyranosyl or mannopyranosyl units are discernible.

### Pretreatment conditions

**Fig. 2:**
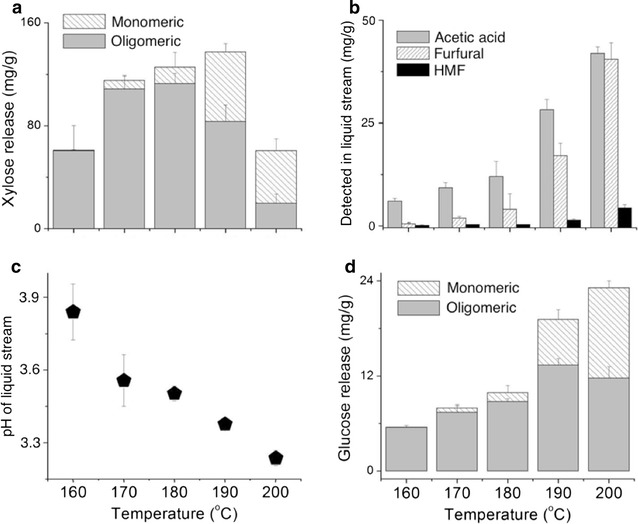
Autohydrolysis of poplar wood at different temperatures showing **a** xylose oligomer and monomer release, **b** acetic acid and sugar degradation products furfural and HMF, **c** pH, and **d** glucose oligomer and monomer release. Samples were pre-incubated at 60 °C for 1 h. Liquid phase was water, 5% solids loading, pretreatment time 60 min. *Error bars* represent standard error of the mean from the average of three replicates

A small-scale dilute-acid-pretreatment method was developed to compare sugar and acetate release among wood samples. A temperature of 180 °C was the chosen reactor temperature because it yielded the highest proportion of oligomeric xylose with the least amount of carbohydrate degradation (Fig. 2). The reaction pressure (200 psi) and temperature (180 °C) in the pressure vessels were comparable to those employed in mainstream dilute acid pretreatment operations [38].

To establish suitable pretreatment conditions, a single large batch of wood flour originating from one sample was subjected to several different pretreatment conditions. Initially, wood samples were pretreated in hot pressurized liquid water to investigate dissolution over different severity gradients facilitated by temperature. Effectively, these preliminary experiments tested the autohydrolysis of poplar wood at temperatures ranging from 160 to 200 °C (Fig. 2). The products that were liberated by the hot-water pretreatment and measured included xylose (Fig. 2a), furfural, hydroxymethylfurfural (HMF) and acetic acid (Fig. 2b), and glucose (Fig. 2d). The pH of the liquid fraction decreased from 3.8 (pretreatment at 160 °C) to 3.2 at 200 °C (Fig. 2c). There was 110 mg/g oligomeric xylose in the liquid stream following pretreatment at 180 °C and, at higher temperatures, this value decreased first to 85 mg/g at 190 °C and then to 20 mg/g at 200 °C. Monomeric xylose was not detected in the liquid phase following hot-water pretreatment at 160 °C for 60 min. At higher temperatures, monomeric xylose increased from 10 mg/g at 170 °C to 15 mg/g at 180 °C and peaked at 50 mg/g at 190 °C. Pretreatment at 200 °C resulted in a decrease in monomeric xylose to ~40 mg/g. Figure 2b shows furfural, a by-product of xylan degradation, sharply increasing with temperature. No furfural was detected in the liquid phase following pretreatment at 160 °C. Furfural increased to 3, 5, 18 and 38 mg/g as pretreatment temperature incrementally increased. There was 6 mg acetic acid per gram of wood in the liquid stream after treatment at 160 °C. This corresponds to approximately 10% of the available acetate in the wood. Pretreatment at 170, 180, 190, and 200 °C yielded 10, 12, 28, and 40 mg/g acetic acid, respectively. There was no HMF detectable until pretreatment at 200 °C, at which temperature 6 mg/g was in the liquid fraction.

Poplar wood was then pretreated at 180 °C in acetic acid (0–9% v/v; Table 2). Total xylose and glucose release increased incrementally with higher concentrations of acetic acid. Values for total effective xylose and glucose release are the sum of monomeric, oligomeric, and dehydrated forms (furfural or HMF). Following 70-min pretreatment at 180 °C in water with 0% acetic acid, 84 mg/g xylose and 13 mg/g glucose formed. This result is comparable to that from the temperature optimization experiment described above. Sugar release was only significantly different between hot pressurized liquid water and 3% acetic acid for monomeric xylose. Following the addition of 6% acetic acid, total effective xylose release increased to 148 mg/g and glucose to 28 mg/g. At 9% acetic acid concentration, xylose and glucose release were 153 and 29 mg/g, respectively. Each 3% increase of acetic acid resulted in an increase in furfural and HMF product.

**Table 2:** Acetic acid catalyzes the hydrolysis of wood polysaccharides during pretreatment

Added acetic acid	Xylose			Total effective xylose release	Glucose			Total effective glucose release
	Monomeric	Oligomeric	Furfural		Monomeric	Oligomeric	HMF	
0.0	4.01 ± 1.42^a^	59.1 ± 7.62^a^	3.61 ± 1.60^a^	84.4 ± 19.1^a^	1.40 ± 0.16^a^	10.2 ± 1.67^a^	0.41 ± 0.08^a^	13.0 ± 0.97^a^
3.2	7.93 ± 1.05^b^	56.3 ± 8.60^a^	10.8 ± 4.84^a^	97.4 ± 18.9^a^	2.52 ± 0.77^a,b^	14.9 ± 2.81^a^	1.16 ± 0.53^a^	19.1 ± 3.95^a,b^
6.3	11.2 ± 2.28^c^	72.1 ± 19.4^a^	21.4 ± 9.12^a^	148 ± 25.9^b^	3.36 ± 0.76^b,c^	19.1 ± 3.85^b^	1.61 ± 0.91^a^	27.7 ± 3.90^b,c^
9.4	9.90 ± 3.60^a^	74.0 ± 22.9^a^	31.0 ± 16.3^a^	153 ± 44.2^b^	2.24 ± 0.87^c^	22.7 ± 2.72^b^	3.14 ± 1.74^a^	29.4 ± 4.23^c^

All values are in milligrams per gram ± standard error of the mean. Total effective sugar release is the sum of all three fractions, with degradation products converted on a molar ratio to xylose. Pretreatments were at 180 °C for 70 min. Superscripts indicate statistical significance at *p* value <0.05

Next, we pretreated poplar wood for varying time periods and sulphuric acid concentrations (without exogenous acetic acid). Twelve “regimes” consisting of 10, 30, or 60 min pretreatment supplemented with 0.0, 0.1, 0.3, or 0.6% (w/w) sulphuric acid were examined. Table 3 shows the percent wood dissolved or degraded versus the percent solid wood residue. Wood dissolution was less than 40 mg/g for regimes 1–5. Thirty-minutes pretreatment using 0.3% catalyst dissolved the same mass of wood as 60 min of uncatalyzed pretreatment (regimes 7 and 9). Thirty-minutes pretreatment using 0.6% catalyst yielded as much sugar as 60-min pretreatment using 0.1% sulphuric acid (regimes 8 and 10). The increase in degradation products at regimes 11 and 12 was accompanied by a decrease in dissolved sugars. Increasing severity further decreased the proportion of oligomers to monomers.

**Table 3:** Dissolution and degradation of *P. trichocarpa* wood at various dilute acid pretreatment regimes

Regime	Time (min)	H_2_SO_4_	Dissolved sugars	Wood residue	Degraded	Xyl O:M	Glc O:M	Acetate			
								AA	XOS	WR	AA:XOS:WR
1	10	0.0	33	930	ND	0.6	1.1	0.6	ND	50	1.0:ND:83
2	10	1.0	34	910	ND	1.8	1.1	1.4	ND	48	1.0:ND:34
3	10	3.0	33	880	ND	7.1	1.0	1.4	ND	50	1.0:ND:36
4	10	6.0	33	880	ND	3.2	1.2	1.6	ND	50	1.0:ND:31
5	30	0.0	38	880	ND	100	2.2	0.6	ND	50	1.0:ND:83
6	30	1.0	140	840	ND	4.5	2.1	3.0	12	41	1.0:4.0:13.7
7	30	3.0	280	740	ND	1.1	1.1	18	15	24	1.2:1.0:1.6
8	30	6.0	380	720	ND	0.5	0.5	37	ND	5.4	6.9:ND:1.0
9	60	0.0	280	720	ND	20	4.8	4.1	21	50	1.0:5.1:12.2
10	60	1.0	370	700	ND	0.4	0.3	40	5.5	3.0	13.3:1.8:1.0
11	60	3.0	340	690	74	0.2	0.2	54	ND	ND	1.0:ND:ND
12	60	6.0	210	680	230	0.8	0.3	58	ND	ND	1.0:ND:ND

Pretreatment temperature was 180 °C. *H*_*2*_ *SO*_*4*_ sulphuric acid, *Glu* glucose, *Xyl* xylose, *O:M* oligomer-to-monomer ratio; Acetate partitions into acetic acid (AA), dissolved acetate on xylooligosaccharides (XOS), and acetate on wood residue (WR). *ND* not detected (less than 5% w/w). Values are the average of three technical replicates. Soluble lignin and minor sugars arabinose, rhamnose and galactose are not included in the mass balance. All values except ratios are mg/g

A key consideration in the selection of pretreatment time and the concentration of sulphuric acid was the partitioning of acetate into its three possible forms: as acetate attached to wood (WR), dissolved and attached to short xylooligosaccharides (XOS), and as acetic acid (AA) (Table 3). As pretreatment severity increased, acetylated xylan hydrolyzed to produce acetylated XOS. Thereafter, these acetylated XOS hydrolyzed to acetic acid and xylose (or low DP XOS). Mild pretreatments resulted in very little acetic acid liberation; harsher pretreatments resulted in high acetic acid concentrations, with very little acetate remaining on XOS or wood. Based on our original mass balance, all acetate in wood hydrolyzed to acetic acid at the highest pretreatment severity. Under these conditions, 60 mg/g acetate was released from wood. We therefore chose suitable pretreatment conditions based on acetate release, as well as carbohydrate solubilization and degradation. Regime 7—pretreatment in 0.3% sulphuric acid catalyst for 30 min—provided the “middle ground” for acetate partitioning whereby acetic acid, acetylated wood, and acetylated XOS were present in approximately equal fractions. Regime 7 dissolved on average 28% (w/w) of wood, including two-thirds of the available xylan and one-twentieth of the available glucan (Table 3).

### Comparing acetate and sugar release in different wood samples

Having established the impact of acetic acid on poplar wood solubilization, we evaluated the impact of native acetate in 19 different poplar wood samples using the sulphuric acid-catalyzed pretreatment regime 7. Samples came from the natural population and had known cell wall chemistries and similar ultrastructural properties (density, fiber dimensions, and crystallinity; data not shown).

Pretreatment sugar release is shown in Table 4. Overall sugar yield and the oligomer-to-monomer ration (O:M) of xylose and glucose varied twofold. Poplar wood samples released 63–184 mg xylose, and 6–22 mg glucose per gram of extractives-free, oven-dried wood. Monomeric xylose release ranged from 3–11 mg/g, whereas oligomeric xylose amounted to 60–140 mg/g. Monomeric glucose release ranged between 0.2 and 1.6 mg/g, whereas oligomeric glucose ranged from 6 to 20 mg/g. Individuals with high xylose release also released high quantities of glucose.

**Table 4:** Xylose, glucose, and acetate release and partitioning following pretreatment

Sample number	Xylose		Glucose		Acetate			
	Total	O:M	Total	O:M	AA	XOS	WR	AA: XOS: WR
1	148 ± 2.0	11.7	21.8 ± 2.0	17.8	13.0 ± 0.4	26.0 ± 0.3	14.4 ± 1.0	1.0: 2.0: 1.1
2	140 ± 14.9	15.2	18.4 ± 3.3	21.2	13.2 ± 2.2	19.9 ± 0.4	19.3 ± 3.5	1.0: 1.5: 1.5
3	136 ± 2.1	16.8	16.7 ± 0.3	23.5	12.7 ± 0.2	22.0 ± 0.9	25.3 ± 8.9	1.0: 1.7: 2.0
4	135 ± 6.2	14.9	15.1 ± 0.5	18.7	12.4 ± 0.6	27.1 ± 1.4	25.8 ± 5.1	1.0: 2.2: 2.1
5	134 ± 8.3	15.9	18.2 ± 1.4	21.7	12.3 ± 0.9	20.0 ± 1.2	20.9 ± 0.3	1.0: 1.6: 1.7
6	129 ± 6.3	14.3	13.2 ± 0.9	14.6	12.7 ± 0.7	17.7 ± 1.2	19.7 ± 2.0	1.0: 1.4: 1.5
7	126 ± 15.1	14.8	15.1 ± 3.4	17.8	11.5 ± 2.8	17.3 ± 1.4	22.2 ± 5.0	1.0: 1.5: 1.9
8	120 ± 14.2	17.7	14.5 ± 2.3	23.1	10.7 ± 1.7	17.0 ± 1.5	25.2 ± 4.7	1.0: 1.6: 2.3
9	118 ± 4.7	16.3	15.8 ± 2.0	20.5	10.4 ± 0.9	22.7 ± 2.3	19.5 ± 1.0	1.0: 2.2: 1.9
10	118 ± 15.9	15.2	15.0 ± 2.5	17.3	10.5 ± 3.8	17.8 ± 2.0	26.2 ± 1.8	1.0: 1.7: 2.5
11	115 ± 17.5	13.6	15.8 ± 2.5	14.9	11.4 ± 2.4	14.6 ± 1.8	26.6 ± 3.1	1.0: 1.3: 2.3
12	115 ± 3.5	15.5	10.0 ± 0.3	18.4	8.7 ± 0.5	15.5 ± 2.0	29.7 ± 5.9	1.0: 1.8: 3.4
13	113 ± 35.2	13.6	16.3 ± 6.4	14.9	12.2 ± 3.6	12.0 ± 1.7	18.9 ± 1.2	1.0: 1.0: 1.5
14	113 ± 3.5	22.9	16.7 ± 0.6	38.9	11.6 ± 0.5	15.8 ± 2.4	24.2 ± 3.6	1.0: 1.4: 2.1
15	108 ± 13.3	14.1	14.6 ± 3.1	14.5	10.7 ± 2.5	18.9 ± 3.0	25.0 ± 2.6	1.0: 1.8: 2.3
16	107 ± 8.6	16.4	10.6 ± 1.1	17.3	10.1 ± 1.2	14.2 ± 2.4	28.7 ± 10.9	1.0: 1.4: 2.8
17	100 ± 22.2	12.5	14.7 ± 2.8	13	9.4 ± 2.8	18.4 ± 3.3	27.9 ± 4.6	1.0: 1.9: 2.9
18	84 ± 5.8	14.9	11.7 ± 0.8	16.2	8.1 ± 0.9	11.5 ± 2.2	29.3 ± 5.3	1.0: 1.4: 3.6
19	63 ± 30.7	12.8	6.3 ± 3.3	13.4	5.6 ± 4.7	ND	34.2 ± 10.7	1.0: ND: 6.1

All values, except ratios, are listed as mg/g ± standard error of the mean. Total xylose and glucose release include the corresponding sugar in its oligomeric and monomeric form. *O:M* oligomer-to-monomer ratio. Acetate partitions into acetic acid (AA), dissolved acetate on xylooligosaccharides (XOS), and acetate on wood residue (WR). *ND* not detected

**Fig. 3:**
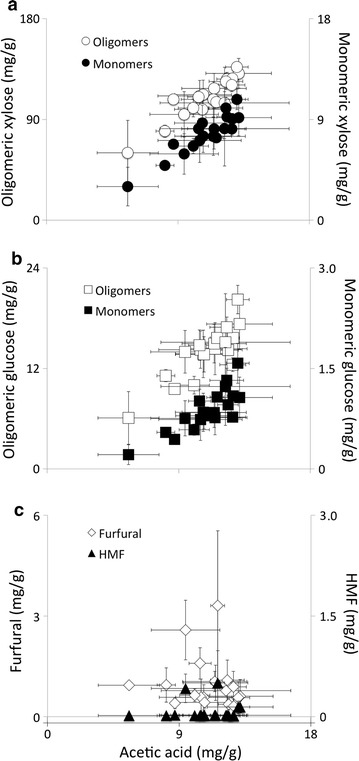
Relationship of acetic acid to **a** xylose, **b** glucose, and **c** degradation products in the pretreatment liquor. *Each marker* represents the average of three technical replicates. *Error bars* show standard error of the mean

Figure 3a shows the relationship between xylose and acetate during pretreatment. There is a strong linear correlation between acetic acid and monomeric xylose (*R* = 0.95). The oligomeric xylose versus acetic acid curve followed a hyperbolic shape that plateaued at 140 mg/g xylose oligomers (Fig. 3a). Oligomeric and monomeric glucose also correlated linearly with acetic acid (*R* = 0.89 and 0.91; Fig. 3b). Figure 3c plots sugar degradation products, HMF and furfural, against acetic acid in pretreatment liquor. Higher acetic acid was not associated with higher furfural or HMF formation.

Table 4 demonstrates how acetate in wood partitioned into three phases. Following pretreatment, it may exist as free acetic acid, or remain linked to dissolved XOS or on wood residues. This acetate partitioning, unique to each sample, suggests a wood chemistry basis for autohydrolysis. If all acetate groups were released in a sample, the resulting solution would be less than 1 mg/g in acetic acid. Sample number 19, for example, had the lowest overall acetate, which partitioned between free acetic acid and wood residue in a 1:6 ratio. In contrast, sample 4 had the highest overall acetate in a 1:2:2 ratio. Acetate levels in samples 16 and 11 were in the middle range, with 1:1:3 and 1:1:2 acetate partitioning, respectively (acetic acid:XOS:solid wood residue). Acetate partitioning clearly reflected different wood chemistries.

### Evaluation of factors affecting acetyl-to-acetic acid hydrolysis

Simple linear regressions can describe acetate partitioning based on other wood chemistry traits (Table 5). Lignin content was inversely correlated with acetate-to-acetic acid hydrolysis (*R* = −0.6, *p* = 0.01). Conversely, there was no significant correlation between native wood cellulose and acetate hydrolyzed (*R* = 0.34, *p* = 0.027). The degree of inherent cell wall acetylation did not correlate with the extent of acetate released during pretreatment (*R* = 0.14, *p* = 0.56), nor did minor variations in starting initial moisture content correlate with acetate-to-acetic acid hydrolysis (*R* = −0.12, *p* = 0.62).

**Table 5:** Possible factors affecting acetyl-to-acetic acid conversion during dilute acid pretreatment of poplar wood

	*R*	*p* value	*n*
Lignin	−0.59	0.010	18
Cellulose	0.34	0.027	18
Total acetate	0.14	0.560	19
Wood moisture	−0.12	0.620	19

*R* Pearson correlation coefficient; *p* value represents the test statistic; *n* number of observations (each is the average of three technical replicates). Cellulose estimates based on glucose content

## Discussion

### Wood sampling and degree of acetylation

In a survey of wood samples from over 200 *P. trichocarpa* accessions growing in a common garden, the overall average degree of acetylation for xylan was approximately 0.6, consistent with earlier findings [13, 39]. In poplar, approximately 90% of acetate is attached to xylopyranosyl units, and it appears that poplar lignin is not significantly acetylated (Fig. 1). The positive relation between xylose and acetate in wood was therefore expected (Table 1), as acetate is added to xylan during xylan biosynthesis in relatively fixed proportions [40].

There is a moderately significant correlation between xylose and acetate content—a trend that may reflect differential degrees of substitution on individual xylopyranosyl units or blocks of xylan. For example, the degree of xylan substitution is not uniform across xylopyranosyl residues. Each xylopyranosyl residue can effectively have zero, one, or two acetate groups decorating the xylose unit. In contrast, some “blocks” of the xylan polymer may have higher localized acetate content relative to others. Another degree of uncertainty in the xylose-acetate trend is the presence of other acetylated polymers in the cell wall. Xyloglucans and glucomannans are additional hemicelluloses in poplar wood that can also be acetylated; however, both of these comprise less than 5% of normal poplar wood composition [9]. These factors explain some of the uncertainty in the xylose-acetate trend recorded in Table 1.

The glucose-acetate trend noted in Table 1 agrees with previously noted relationships between xylose and cellulose in *P. trichocarpa* [7]. Table 1 records statistically significant trends between acetate and cell wall carbohydrates: arabinose, rhamnose, galactose, and mannose. It could be that hemicellulose acetylation couples with the deposition of other cell wall components such as pectin. Acid-soluble lignin and acetate deposit in related amounts, but this is not the case with acid-insoluble lignin (Table 1). Acid-soluble lignins possess ether-linkages and this could indicate an association with cell wall acetate. The bulk of lignin (acid-soluble, >80% w/w) does not correlate with acetate content (Table 1). This could be due to the timed deposition of secondary-wall-specific lignin versus acetate in the secondary cell wall. Lignification occurs after acetylated xylans are synthesized and shuttled to the apoplast [41].

In this study of 200 individual poplar trees representing over 100 unrelated genotypes, there was no link between wood acetate and total biomass accrued (Table 1). This trait independence implies that possibilities for selectively breeding for wood acetate while maintaining biomass yield is indeed possible [42].

### Dilute acid pretreatment of poplar wood

Table 2 shows that pretreating poplar wood in acetic acid increases its solubilization. Acetic acid alone was effective in catalyzing glucose and xylose release, i.e., that acetic acid’s organosolv capacity is expeditious for sugar release. For example, 4% acetic acid or above facilitates 5% lignin removal during hardwood pretreatment [43]. In another study, acetic acid boosted glucan and xylan yields by up to 50% in natural *Populus* variants [44]. As with other acid catalysts, excessively high concentrations of acetic acid result in yield loss from degradation. Each 3% increment of acetic acid resulted in a twofold increase in furfural products. The buildup of undesirable furfural is preventable using a flow-through system, where excess acetic acid originating from the wood would be controlled [48].

Previous studies have shown that endogenous acetic acid can catalyze the breakdown of hemicelluloses, depending on the pretreatment employed for processing the biomass [23, 24, 43, 45–47]. However, there has been no quantification of the proportion of acetate converted to acetic acid. Cell wall chemistry and ultrastructure affect the proportion of acetate release; this was demonstrated when wheat straw released one-third as much acetic acid as hybrid poplar chips following the same pretreatment [39]. The goal of our next analysis was to pinpoint these components. Table 4 presents data for acetate partitioning in wood samples varying in cell wall chemistry, and Table 5 ties these data in regression analyses to cellulose, lignin, acetate, and the moisture content of wood. Nineteen distinct *P. trichocarpa* genotypes were pretreated identically.

Figure 3 shows that, at the selected pretreatment condition, acetic acid did not correlate with the degradation of carbohydrates into HMF and furfural. Instead, HMF and furfural formation likely depended on sulfuric acid concentrations [20]. Therefore, in order to optimize sugar release, high levels of inherent acetic acid are desirable in the pretreatment, as they correlate well with sugar release but not degradation, as is consistent with prior findings [48].

Table 4 records levels of acetate bound to xylan on wood residue, dissolved in solution on XOS, or hydrolyzed to acetic acid. The absence of acetate on xylose oligosaccharides in sample 15 implies that the formation of acetic acid can precede xylan hydrolysis during pretreatment. Xylose monomers may be formed either by direct degradation of xylan in the wood or by depolymerization of the solubilized oligomeric xylan [49]. High-pressure environments could limit acetate hydrolysis; the formation of volatiles such as acetic acid occurs less prominently in high-pressure reactions [50]. Moreover, dilute acid pretreatment on hardwood at atmospheric pressure completely removed acetate groups [51].

Under the mild pretreatment conditions employed, acetate partitioning is useful for studying wood deconstruction. A high pretreatment severity would result in all acetate groups being hydrolyzed to acetic acid (Table 3). In the present pretreatment, one-sixth to one-third of acetate groups were hydrolyzed to acetic acid (Table 4). Examining the partitioning of acetate into the three different pools, it is apparent that a higher proportion of acetate is retained in the wood residue than in the dissolved fraction. This suggests that less-acetylated xylan is more easily removed from the secondary cell wall during pretreatment, and leads to the interpretation that cell wall acetylation could be a factor distinguishing slow- and fast-reacting xylan [52]. Table 4 suggests that fast-reacting xylan has a degree of acetylation of 0.35, and slow-reacting xylan has a degree of acetylation of 0.73. Previous studies have speculated that slow-reacting xylan retains its acetate substituents and is “contaminated with” or “embedded within” lignin [22, 53]. In addition, wood samples with a higher total amount of acetate did not release the highest amount of acetic acid. This implies that there are additional factors limiting the removal of highly acetylated xylan from the cell wall.

### Factors affecting xylan dissolution

Our observations show that wood with higher lignin content generally released less xylan and acetic acid (Table 5), ultimately decreasing pretreatment sugar yield. This finding agrees with Timmel [54], who compared xylan removal in aspen and elm wood. Elm contains 15% more lignin than aspen and treatment with aqueous potassium hydroxide removed the entire proportion of aspen xylan, but only one-fourth that in elm. An explanation for these results is two-tiered: First, lignin retains xylan in the wood residue by non-covalent interactions and, second, dissolved lignin interferes with xylan dissolution [55]. Poplar xylan has high acetate content; thus, the effect of lignin on wood recalcitrance is more pronounced than in other feedstocks such as sugarcane bagasse [56].

Recalcitrance of xylan to pretreatment depends upon non-covalent or covalent interactions as well as mechanical entanglement of xylan with itself or other cell wall polymers [57]. The findings of the current study suggest that lignin and xylan interact, and that acetate content influences the interaction between these two major cell wall polymers. The amount of acetate hydrolyzed inversely correlates with total lignin content (*R* = −0.6; Table 5), and this supports previous findings that lignin increases biomass recalcitrance to pretreatment [58]. That acetylated xylan forms complexes with lignin in aqueous pretreatment slurries can be explained by hydrophobic effects. In sufficient quantity, hydrophobic or van der Waals interactions can facilitate intermolecular adhesion in the secondary cell wall [55, 59]. The removal of acetate from solid wood residue and their dissolution as XOS could be enthalpy-driven and highly affected by non-covalent interactions.

## Conclusions

Acetate endogenous to woody biomass could improve sugar release during pretreatment, an effect also noted by Ewanick et al. [60]. Acetate hydrolysis did not vary with wood cellulose content, which is consistent with the hypothesis that interactions between acetate and the cellulose microfibril are minimal [61]. Results from this and other studies also show that some acetate is retained in xylan in woody biomass during pretreatment [62]. Maximizing acetate release may be one way to increase sugar yield without increasing sugar degradation (Fig. 3). For example, acetate tends to be associated with slow-reacting xylans more than fast-reacting xylans (Table 4). Finally, there is more acetic acid in the liquid stream following pretreatment of lower-lignin wood samples (Table 5). Tailoring pretreatments by taking into consideration these compositional relationships could increase the effectiveness of biomass refining processes.

This study provides insight into the deconstruction of *P. trichocarpa* during pretreatment. The catalytic potential of acetic acid released from the cell wall is controllable by altering reaction time and changing acid concentration. Strong correlations among woods from 19 individuals suggest that acetate aids in dissolving hemicelluloses during pretreatment, but only if hydrolyzed to free acetic acid. Our findings suggest that highly acetylated xylan is more difficult to remove from wood samples, as feedstocks with higher lignin released less acetic acid into solution. This relates the catalytic effect of acetate in poplar wood to its lignin content. These findings demonstrate the importance of considering cell wall acetate when evaluating potential bioenergy crops. As acetylated xylan is the major hemicellulose present in the secondary xylem of most dicot species, this knowledge is applicable to other lignocellulosic biofuel feedstocks, such as shrub willow and eucalyptus.

## Methods

### Wood processing and compositional analysis

*Populus balsamifera* subsp. *trichocarpa* individuals were grown in a common garden established by the British Columbia Ministry of Forests at the University of British Columbia [63]. Two hundred of 500 available individuals planted in Totem Field in June 2008 were harvested in March 2012 according to McKown et al. [64]. Cookies were cut 6″ from the base, and wood was processed to remove bark and pith. The wood specimens were then ground in a Wiley-mill fit with 40-mesh screen and divided into technical replicates. Samples were stored at −20 °C until subjected to pretreatment.

Wood composition was determined by two-stage acid hydrolysis (Klason method), according to Cullis [65]. Briefly, 3 mL of 72% sulphuric acid was added to 200 mg of extractives-free ground wood in a reaction flask at room temperature. The reaction was stirred every 3 min for 2 h. Nanopure water was used to dilute the reaction to a final sulphuric acid concentration of 4%. Carbohydrates were hydrolyzed in an autoclave at 121 °C for 1 h. High-performance anion-exchange liquid chromatography quantified constituent sugars. The neutral sugars separated on a Carbopak-PA1 anion-exchange resin using an AS50 autosampler, a GS50 gradient pump, and an ED50 electrochemical detector (Dionex, USA). Isocratic elution in deionized water occurred over 35 min; next, a linear gradient ramping to 0.5 M NaOH for 10 min washed out strongly adsorbing components. From 45–60 min, a mobile phase of pure water equilibrated the resin for the next injection. Peaks were manually integrated and quantified against sugar standards. Molar stoichiometrics accounted for mass loss following the hydrolysis of polysaccharides into monosaccharides (0.90 for hexose sugars, 0.88 pentose sugars), and acetic acid to acetate (0.98). Lignin was recovered as acid-insoluble (Klason) and acid-soluble lignin. Acid-insoluble lignin was the hydrolysate retentate in a medium-coarseness sintered glass crucible. Acid-soluble lignin in the filtrate was estimated by Beer–Lambert’s Law using an absorbance at 205 nm and extinction coefficient of *ε* = 110 L/g cm [66].

To quantify acetate, a saponification reaction from Browning [67] was conducted. Sodium hydroxide (0.2 M) reacted with acetone-extracted wood samples at a 2% solids loading. The reaction incubated at 120 °C with constant shaking (500 rpm) for 75 min. Sulphuric acid (72% w/w) acidified each sample to pH 2 ± 1 and cooling took place in an ice bath for 5 min. Centrifuging samples at 13,000*g* for 2 min separated solid and liquid phases. The supernatant eluted through a 0.45 μm filter into a 2-mL glass vial. Samples were injected onto an HPX-87H column (Aminex, USA) on an high-pressure liquid chromatography instrument equipped with an ASI-100 Autosampler, a P60 HPLC Quaternary Gradient Pump, and a PDA-100 photodiode array detector set to 205 nm (Dionex, USA). The mobile phase was 5 mM sulphuric acid at a flow rate of 0.7 mL/min. Acetic acid peaks were integrated manually and their areas measured against standards of known concentration.

### NMR

NMR analysis was performed as described in the recent protocol [68]. Briefly, plant biomass was air-dried to a constant moisture content and cryogenically pre-ground for 2 min at 30 Hz using a Retsch (Newtown, PA, USA) MM301 mixer mill. The pre-ground cell walls were extracted with distilled water, followed by 80% ethanol, using ultrasonication. Isolated cell walls (200 mg) were then finely milled using a Retsch PM100 planetary ball mill for 70 in 10-min intervals with 5-min interval breaks. Approximately 30–60 mg of extractives-free, ball-milled plant cell wall material was transferred to a 5-mm NMR tube, and 500 μL of premixed DMSO-d_6_/pyridine-d_5_ (4:1) was added directly into the NMR tube containing individual samples. The NMR solvent mixture was carefully introduced (via a syringe), spreading it from the bottom of the NMR tube, along the sides, and towards the top of the sample. The NMR tubes were then placed in an ultrasonic bath and sonicated for 1–5 h, until the gel became homogeneous; the final sample height in the tube was ~4 to 5 cm. 2D ^1^H–^13^C HSQC spectra were acquired using a standard Bruker pulse program (hsqcetgpsisp2.2). The NMR spectra had the following parameters typical for plant cell wall samples: spectra were acquired from 10 to 0 ppm in *F*2 (^1^H) using 2800 data points for an acquisition time (AQ) of 200 ms, an interscan delay (*D*1) of 1 s, 200–0 ppm in F1 (^13^C) using 560 increments (*F*1 acquisition time 8 ms) of 56 scans, with a total acquisition time of 11 h. Processing used typical matched Gaussian apodization in *F*2 and squared cosine-bell in *F*1. Interactive integrations of contours in 2D HSQC plots were carried out using Bruker’s TopSpin 3.5 (Mac) software, as was all data processing.

### Pretreatment of wood—autohydrolysis, acetic-acid-catalyzed, and sulphuric-acid-catalyzed

Wood flour and pretreatment liquid (dilute water, acetic or sulphuric acid) were added into reactors at 5% (w/w) solids loading. Reaction vessels with a capacity of 15 mL were stainless steel cylinders with a bolt-screw fitting on either end. Prior to closing the reactors, both fittings were sealed with polytetrafluoroethylene tape. The reactors were vortexed and preincubation at 60 °C for 60 min to ensure impregnation of the wood sample with pretreatment solvent followed. Non-isothermal pretreatment was conducted in a Lindberg Blue M laboratory gravity oven (Thermo Scientific, USA) at 180 °C. At time zero, heating in the oven began. Pressure inside the reactor was measured by attaching a 2000 psi pressure gauge (Ashcroft, USA) to a bolt-screw fitting via 30 cm of stainless steel tubing. All reactions were quenched in an ice bath.

### Analysis of solid and liquid phases after autohydrolysis

Following reactions, the pretreatment liquors and wood residue were transferred to polypropylene tubes wrapped in aluminum foil to prevent furan degradation by ultraviolet light. Samples were stored at 4 °C for a maximum of three days prior to HPLC analysis. Monosaccharides were quantified by high-performance anion-exchange chromatography columns as described above. Oligosaccharides underwent secondary acid hydrolysis, where the original reaction hydrolysates were autoclaved in 2.5% (w/w) sulphuric acid at 121 °C for 60 min, and the carbohydrates were again quantified using high-performance anion-exchange chromatography, and the difference between total sugars in the secondary hydrolysates and monosaccharides in the original reaction hydrolysate was determined to be the oligosaccharide fraction. Separation of the acetic acid, furfural and 5-hydroxymethyl-2-furaldehyde (hydroxymethylfurfural, HMF) was achieved on an Aminex HPX-87H column (BioRad, USA) as described above. After washing, acetate in wood residue was quantified by saponification as described above. Acid-soluble sugars in wood residue were determined using the Klason method described above.

## Additional file

**Additional file 1: Table S1.** Data used to calculate associations presented in Table 1. All values except biomass are in percent extractives-free dry weight. Biomass is in kilograms.

## Abbreviations

AA: acetic acid
DP: degree of polymerization
XOS: xylooligosaccharide
R: Pearson’s correlation coefficient
w/w: weight over weight
v/v: volume over volume
HPLC: high-performance liquid chromatography
HMF: 5-hydroxymethyl-2-furaldehyde
O:M: oligomer-to-monomer ratio
SD: standard deviation
WR: wood residue
