# Supplementary material for: Natural acetylation impacts carbohydrate recovery during deconstruction of Populus trichocarpa wood
Source: Biotechnol Biofuels. 2017 Feb 23;10:48. doi: 10.1186/s13068-017-0734-z (PMC5322675; doi:10.1186/s13068-017-0734-z)
Supplement: Supplementary file 1 — Additional file 1: Table S1. Data used to calculate associations presented in Table 1. All values except biomass are in percent extractives-free dry weight. Biomass is in kilograms. [file 13068_2017_734_MOESM1_ESM.docx]

Table S1. Data used to calculate associations presented in Table 1. All values except biomass are in percent extractives-free dry weight. Biomass is in kilograms.

| Acetate | Glc | Xylose | Lignin (acid-soluble) | Man | Rha | Gal | Lignin (acid-insoluble) | Biomass | Ara |
| --- | --- | --- | --- | --- | --- | --- | --- | --- | --- |
| 3.56 | 56.25 | 15.06 | ND | ND | ND | ND | ND | 2.50 | 0.49 |
| 4.24 | 54.25 | 15.47 | 2.52 | 1.95 | 0.05 | 0.64 | 16.54 | 7.08 | 0.32 |
| 4.32 | 54.56 | 14.89 | 2.15 | 1.55 | 0.16 | 0.68 | 15.97 | 12.84 | 0.33 |
| 4.37 | 49.16 | 16.92 | 2.76 | 2.22 | 0.16 | 0.54 | 17.38 | 18.96 | 0.37 |
| 4.41 | 48.17 | 15.04 | 2.33 | 1.96 | 0.31 | 0.70 | 18.74 | 9.07 | 0.44 |
| 4.45 | 42.07 | 19.63 | 3.46 | 2.97 | 0.30 | 0.47 | 20.63 | 2.22 | 0.48 |
| 4.48 | 45.76 | 17.69 | 2.86 | 2.52 | 0.12 | 0.43 | 20.75 | 1.50 | 0.35 |
| 4.52 | 51.11 | 16.94 | 2.77 | 3.25 | 0.45 | 0.78 | 19.82 | 2.27 | 0.50 |
| 4.53 | 43.96 | 14.68 | 2.64 | 1.88 | 0.26 | 0.50 | 18.85 | 30.75 | 0.32 |
| 4.56 | 54.75 | 19.33 | 2.50 | 2.40 | 0.05 | 0.52 | 17.77 | 29.89 | 0.38 |
| 4.60 | ND | ND | ND | ND | ND | ND | ND | 12.93 | ND |
| 4.61 | 45.59 | 16.73 | 2.85 | 2.16 | 0.08 | 0.40 | 18.32 | 17.42 | 0.38 |
| 4.61 | 49.19 | 16.68 | 2.87 | 2.22 | 0.38 | 0.67 | 20.91 | 7.48 | 0.41 |
| 4.63 | 50.71 | 15.52 | 2.62 | 2.24 | 0.02 | 0.62 | 16.36 | 6.17 | 0.31 |
| 4.63 | 50.62 | 16.74 | 2.49 | 1.78 | 0.06 | 0.50 | 17.22 | 7.98 | 0.35 |
| 4.65 | 48.90 | 16.17 | 2.77 | 2.17 | 0.16 | 0.57 | 18.48 | 9.07 | 0.35 |
| 4.66 | 54.91 | 19.94 | 2.06 | 2.25 | 0.12 | 0.60 | 17.57 | 31.21 | 0.38 |
| 4.67 | ND | ND | ND | ND | ND | ND | ND | 14.38 | ND |
| 4.71 | ND | ND | ND | ND | ND | ND | ND | 8.03 | ND |
| 4.71 | 41.35 | 17.27 | 2.15 | 2.51 | 0.39 | 0.55 | 23.36 | 16.42 | 0.43 |
| 4.71 | ND | ND | ND | ND | ND | ND | ND | 19.64 | ND |
| 4.72 | 51.70 | 16.16 | 2.30 | 1.78 | 0.05 | 0.57 | 15.69 | 13.15 | 0.44 |
| 4.73 | 48.24 | 18.57 | 3.17 | 3.10 | 0.10 | 0.38 | 17.03 | 32.34 | 0.35 |
| 4.73 | 43.17 | 15.48 | 2.74 | 2.45 | 0.10 | 0.49 | 21.90 | 7.94 | 0.36 |
| 4.74 | 53.14 | 17.54 | 2.49 | 2.11 | 0.19 | 0.61 | 16.47 | 26.31 | 0.40 |
| 4.75 | 46.98 | 19.37 | 2.92 | 2.44 | 0.38 | 0.59 | 24.67 | 13.34 | 0.49 |
| 4.76 | 51.76 | 17.82 | 2.55 | 2.67 | 0.10 | 0.57 | 15.46 | 15.88 | 0.36 |
| 4.76 | 40.05 | 18.16 | 2.67 | 2.79 | 0.16 | 0.35 | 19.52 | 13.56 | 0.37 |
| 4.76 | 45.73 | 19.88 | 2.92 | 2.59 | 0.18 | 0.43 | 19.12 | 10.57 | 0.38 |
| 4.77 | 42.19 | 17.77 | 3.26 | 3.57 | 0.34 | 0.46 | 22.06 | 12.02 | 0.42 |
| 4.78 | 45.26 | 15.78 | 3.38 | 2.36 | 0.31 | 0.55 | 18.68 | 8.80 | 0.35 |
| 4.79 | 47.10 | 14.69 | 2.52 | 2.30 | 0.05 | 0.36 | 19.78 | 8.57 | 0.27 |
| 4.80 | ND | ND | ND | ND | ND | ND | ND | 16.28 | ND |
| 4.80 | 45.44 | 19.07 | 2.22 | 2.58 | 0.29 | 0.59 | 19.64 | 7.57 | 0.52 |
| 4.81 | 46.81 | 18.68 | 3.03 | 3.21 | 0.38 | 0.61 | 15.98 | 14.29 | 0.46 |
| 4.82 | 46.98 | 20.13 | 3.29 | 3.17 | 0.23 | 0.39 | 19.09 | 1.18 | 0.41 |
| 4.82 | ND | ND | ND | ND | ND | ND | ND | 25.40 | ND |
| 4.84 | 47.40 | 16.32 | 2.40 | 2.10 | 0.11 | 0.45 | 18.62 | 24.95 | 0.35 |
| 4.84 | 44.62 | 16.63 | 3.07 | 2.85 | 0.06 | 0.38 | 21.72 | 10.21 | 0.35 |
| 4.87 | 52.60 | 16.92 | 2.44 | 1.94 | 0.28 | 0.70 | 15.21 | 15.92 | 0.42 |
| 4.88 | 43.71 | 18.41 | 2.68 | 3.11 | 0.34 | 0.47 | 20.31 | 11.11 | 0.41 |
| 4.88 | ND | ND | ND | ND | ND | ND | ND | 10.70 | ND |
| 4.88 | 51.05 | 17.44 | 2.75 | 2.19 | 0.32 | 0.51 | 17.39 | 33.48 | 0.38 |
| 4.88 | 43.08 | 18.35 | 3.32 | 1.51 | 0.14 | 0.36 | 22.00 | 24.72 | 0.41 |
| Acetate | Glc | Xylose | Lignin (acid-soluble) | Man | Rha | Gal | Lignin (acid-insoluble) | Biomass | Ara |
| 4.89 | ND | ND | ND | ND | ND | ND | ND | 8.48 | ND |
| 4.89 | 52.09 | 20.98 | 3.24 | 3.15 | 0.03 | 0.57 | 17.75 | 21.77 | 0.56 |
| 4.89 | 44.85 | 16.96 | 2.70 | 2.37 | 0.02 | 0.34 | 18.23 | 25.08 | 0.35 |
| 4.90 | 49.23 | 17.75 | 2.81 | 2.79 | 0.41 | 0.73 | 18.88 | 14.06 | 0.37 |
| 4.91 | 49.93 | 17.13 | 2.86 | 2.58 | 0.34 | 0.60 | 20.69 | 7.76 | 0.41 |
| 4.92 | 46.53 | 16.41 | 2.84 | 1.87 | 0.07 | 0.37 | 17.36 | 6.53 | 0.32 |
| 4.92 | 43.91 | 18.92 | 2.81 | 2.83 | 0.37 | 0.55 | 17.75 | 11.11 | 0.40 |
| 4.92 | 46.54 | 17.74 | 3.06 | 2.20 | 0.33 | 0.67 | 18.74 | 7.17 | 0.46 |
| 4.93 | 49.94 | 16.16 | 3.13 | 3.30 | 0.42 | 0.61 | 20.94 | 17.37 | 0.44 |
| 4.93 | 44.95 | 16.41 | 3.26 | 2.61 | 0.10 | 0.45 | 20.54 | 5.81 | 0.36 |
| 4.94 | 43.88 | 17.68 | 2.97 | 2.71 | 0.21 | 0.38 | 21.88 | 5.31 | 0.45 |
| 4.95 | 42.19 | 15.31 | 2.52 | 2.08 | 0.09 | 0.43 | 19.32 | ND | 0.29 |
| 4.95 | ND | ND | ND | ND | ND | ND | ND | 15.88 | ND |
| 4.96 | 50.59 | 17.99 | 2.80 | 1.82 | 0.37 | 0.68 | 15.62 | 8.21 | 0.38 |
| 4.97 | 45.58 | 17.62 | 2.89 | 2.19 | 0.36 | 0.52 | 17.85 | 10.48 | 0.45 |
| 4.97 | ND | ND | ND | ND | ND | ND | ND | 8.98 | ND |
| 4.98 | 45.99 | 16.36 | 3.05 | 2.31 | 0.08 | 0.48 | 18.94 |  | 0.33 |
| 4.99 | 46.06 | 16.91 | 3.21 | 3.04 | 0.34 | 0.54 | 20.96 | 5.85 | 0.40 |
| 4.99 | ND | ND | ND | ND | ND | ND | ND | 1.59 | ND |
| 5.00 | 49.87 | 19.87 | 2.95 | 3.16 | 0.46 | 0.60 | 17.70 | 27.53 | 0.47 |
| 5.00 | 46.76 | 22.89 | 2.70 | 2.96 | 0.44 | 0.51 | 22.47 | 19.91 | 0.43 |
| 5.01 | 47.50 | 18.82 | 2.41 | 2.91 | 0.24 | 0.47 | 19.47 | 23.13 | 0.43 |
| 5.01 | ND | ND | ND | ND | ND | ND | ND | 10.84 | ND |
| 5.01 | 43.20 | 16.54 | 2.90 | 2.63 | 0.15 | 0.38 | 18.78 | 11.66 | 0.34 |
| 5.01 | 43.53 | 18.04 | 3.08 | 2.76 | 0.11 | 0.34 | 18.05 | 7.35 | 0.37 |
| 5.02 | ND | ND | ND | ND | ND | ND | ND | 14.06 | ND |
| 5.02 | 47.20 | 17.72 | 2.96 | 2.87 | 0.39 | 0.69 | 20.36 | 1.77 | 0.41 |
| 5.05 | 41.47 | 17.86 | 3.32 | 2.77 | 0.09 | 0.37 | 22.56 | 12.34 | 0.30 |
| 5.05 | 45.19 | 19.89 | 3.00 | 2.83 | 0.28 | 0.42 | 17.38 | 7.85 | 0.37 |
| 5.06 | 47.60 | 18.37 | 2.73 | 2.38 | 0.17 | 0.43 | 17.67 | 19.28 | 0.36 |
| 5.06 | 48.35 | 16.55 | 2.78 | 2.00 | 0.19 | 0.45 | 16.28 | 14.51 | 0.31 |
| 5.06 | 44.27 | 17.40 | 2.82 | 2.14 | 0.24 | 0.41 | 16.71 | ND | 0.38 |
| 5.06 | 47.77 | 18.87 | 2.89 | 2.89 | 0.17 | 0.44 | 17.19 | 10.98 | 0.40 |
| 5.06 | 49.83 | 17.34 | 3.09 | 3.11 | 0.44 | 0.72 | 17.58 | 5.99 | 0.49 |
| 5.06 | 42.60 | 17.53 | 2.48 | 2.65 | 0.39 | 0.55 | 19.92 | 14.42 | 0.55 |
| 5.07 | 43.74 | 18.18 | 2.70 | 3.18 | 0.21 | 0.39 | 20.51 | 5.53 | 0.36 |
| 5.07 | 47.19 | 18.14 | 2.63 | 2.70 | 0.25 | 0.40 | 17.35 | 3.86 | 0.35 |
| 5.07 | 43.30 | 19.49 | 2.95 | 2.57 | 0.37 | 0.53 | 19.72 | 4.58 | 0.48 |
| 5.08 | 49.65 | 20.60 | 2.58 | 3.90 | 0.10 | 0.40 | 19.72 | 11.79 | 0.40 |
| 5.08 | 48.07 | 18.98 | 2.89 | 3.55 | 0.49 | 0.59 | 19.14 | 13.83 | 0.50 |
| 5.08 | 47.89 | 20.02 | 3.07 | 2.51 | 0.36 | 0.57 | 16.47 | ND | 0.48 |
| 5.09 | 44.48 | 19.64 | 2.86 | 2.83 | 0.13 | 0.44 | 23.49 | 26.76 | 0.35 |
| 5.09 | 44.00 | 18.43 | 2.81 | 2.74 | 0.37 | 0.49 | 22.43 | 6.76 | 0.43 |
| 5.09 | 54.19 | 20.43 | 2.44 | 2.89 | 0.50 | 0.64 | 17.72 | 14.42 | 0.51 |
| 5.10 | 43.99 | 16.65 | 2.94 | 1.81 | 0.22 | 0.36 | 18.62 | 3.27 | 0.33 |
| Acetate | Glc | Xylose | Lignin (acid-soluble) | Man | Rha | Gal | Lignin (acid-insoluble) | Biomass | Ara |
| 5.10 | 43.13 | 17.80 | 2.68 | 3.00 | 0.06 | 0.33 | 21.97 | 7.53 | 0.41 |
| 5.11 | 46.13 | 19.27 | 3.26 | 2.97 | 0.13 | 0.40 | 18.14 | 1.86 | 0.30 |
| 5.11 | 44.95 | 18.64 | 2.93 | 2.68 | 0.10 | 0.38 | 17.73 | 4.67 | 0.35 |
| 5.11 | 46.67 | 17.76 | 2.88 | 2.37 | 0.09 | 0.45 | 17.27 | 6.80 | 0.40 |
| 5.12 | 45.63 | 14.90 | 3.72 | 1.84 | 0.43 | 0.67 | 16.63 | 5.49 | 0.55 |
| 5.12 | 49.86 | 17.98 | 3.29 | 2.38 | 0.28 | 0.51 | 18.47 | 9.57 | 0.34 |
| 5.12 | 41.86 | 18.55 | 3.04 | 2.61 | 0.16 | 0.39 | 18.90 | 14.15 | 0.37 |
| 5.13 | 47.26 | 21.38 | 3.30 | 2.89 | 0.47 | 0.62 | 20.05 | 5.67 | 0.49 |
| 5.13 | 40.50 | 17.64 | 3.16 | 2.92 | 0.37 | 0.43 | 22.09 | 8.30 | 0.41 |
| 5.13 | 44.98 | 19.01 | 3.17 | 2.14 | 0.36 | 0.53 | 20.42 | 9.71 | 0.37 |
| 5.13 | ND | ND | ND | ND | ND | ND | ND | 14.65 | ND |
| 5.13 | 44.40 | 18.99 | 2.87 | 2.63 | 0.23 | 0.45 | 20.23 | 18.33 | 0.52 |
| 5.13 | 40.81 | 17.56 | 3.21 | 2.55 | 0.35 | 0.45 | 17.45 | 21.50 | 0.40 |
| 5.14 | 43.45 | 16.20 | 3.52 | 3.14 | 0.15 | 0.43 | 19.15 | 2.54 | 0.33 |
| 5.14 | 45.72 | 18.38 | 2.45 | 3.10 | 0.35 | 0.54 | 18.03 | 26.72 | 0.38 |
| 5.15 | 49.36 | 20.52 | 2.86 | 3.98 | 0.27 | 0.51 | 19.96 | 7.26 | 0.38 |
| 5.15 | 46.68 | 20.39 | 2.99 | 1.63 | 0.14 | 0.45 | 21.04 | 8.39 | 0.38 |
| 5.15 | 41.34 | 16.61 | 2.96 | 2.76 | 0.22 | 0.46 | 19.67 | 0.36 | 0.39 |
| 5.15 | 48.10 | 21.67 | 3.35 | 3.56 | 0.15 | 0.52 | 19.40 | 6.71 | 0.47 |
| 5.15 | 46.24 | 18.29 | 2.95 | 1.33 | 0.05 | 0.37 | 18.82 | 14.38 | 0.37 |
| 5.15 | 49.59 | 21.35 | 2.70 | 3.95 | 0.21 | 0.48 | 16.36 | 8.94 | 0.44 |
| 5.15 | 46.00 | 14.57 | 2.97 | 2.18 | 0.14 | 0.52 | 16.62 | 27.99 | 0.41 |
| 5.16 | 44.95 | 16.58 | 3.07 | 2.48 | 0.33 | 0.56 | 17.83 | 9.62 | 0.39 |
| 5.16 | ND | ND | ND | ND | ND | ND | ND | 14.56 | ND |
| 5.16 | 42.71 | 13.15 | 3.49 | 2.21 | 0.26 | 0.52 | 21.97 | 3.18 | 0.27 |
| 5.17 | ND | ND | ND | ND | ND | ND | ND | 12.25 | ND |
| 5.18 | 48.52 | 20.76 | 3.06 | 2.67 | 0.41 | 0.60 | 20.35 | 10.25 | 0.45 |
| 5.19 | 41.92 | 16.41 | 2.73 | 2.53 | 0.10 | 0.31 | 21.91 | 11.70 | 0.28 |
| 5.19 | 42.80 | 16.95 | 2.72 | 2.61 | 0.13 | 0.45 | 21.29 | 13.29 | 0.37 |
| 5.19 | 42.20 | 18.72 | 3.15 | 2.87 | 0.10 | 0.32 | 20.39 | 20.96 | 0.35 |
| 5.19 | 45.69 | 17.57 | 2.90 | 2.80 | 0.16 | 0.38 | 20.41 | 22.36 | 0.32 |
| 5.20 | 45.48 | 19.21 | 3.09 | 2.84 | 0.20 | 0.42 | 16.97 | 27.22 | 0.36 |
| 5.20 | 45.72 | 16.82 | 2.63 | 1.70 | 0.38 | 0.64 | 17.07 | 13.20 | 0.34 |
| 5.20 | 48.63 | 18.26 | 2.63 | 2.60 | 0.14 | 0.45 | 17.12 | 7.17 | 0.32 |
| 5.20 | ND | ND | ND | ND | ND | ND | ND | 7.94 | ND |
| 5.20 | 44.01 | 19.44 | 3.03 | 1.48 | 0.19 | 0.39 | 24.18 | 18.69 | 0.39 |
| 5.20 | 51.12 | 19.29 | 2.74 | 1.31 | 0.11 | 0.47 | 20.29 | 15.65 | 0.33 |
| 5.21 | 44.63 | 19.05 | 2.91 | 2.86 | 0.20 | 0.39 | 19.06 | 6.62 | 0.33 |
| 5.22 | 41.10 | 17.46 | 3.32 | 2.86 | 0.21 | 0.36 | 19.20 | 2.54 | 0.42 |
| 5.22 | ND | ND | ND | ND | ND | ND | ND | 13.02 | ND |
| 5.22 | 46.21 | 22.67 | 3.15 | 3.55 | 0.37 | 0.53 | 15.69 | 11.07 | 0.49 |
| 5.23 | 44.79 | 19.50 | 2.62 | 3.26 | 0.07 | 0.37 | 21.54 | 15.83 | 0.44 |
| 5.23 | 42.63 | 16.25 | 2.63 | 2.55 | 0.32 | 0.44 | 19.93 | 3.08 | 0.39 |
| 5.23 | 46.89 | 17.89 | 3.09 | 2.76 | 0.41 | 0.85 | 21.83 | 11.84 | 0.76 |
| 5.24 | 47.76 | 19.50 | 3.58 | 3.00 | 0.12 | 0.56 | 18.34 | 12.25 | 0.38 |
| Acetate | Glc | Xylose | Lignin (acid-soluble) | Man | Rha | Gal | Lignin (acid-insoluble) | Biomass | Ara |
| 5.24 | 44.91 | 13.45 | 2.46 | 1.70 | 0.14 | 0.38 | 17.76 | 10.61 | 0.31 |
| 5.24 | 44.41 | 20.52 | 3.01 | 3.26 | 0.10 | 0.37 | 18.90 | 12.93 | 0.32 |
| 5.24 | 44.94 | 19.50 | 3.05 | 2.96 | 0.50 | 0.38 | 20.96 | 6.89 | 0.38 |
| 5.24 | 46.63 | 19.30 | 3.17 | 3.36 | 0.11 | 0.60 | 21.30 | 9.16 | 0.55 |
| 5.26 | ND | ND | ND | ND | ND | ND | ND | 2.18 | ND |
| 5.26 | 41.06 | 15.84 | 3.20 | 2.53 | 0.18 | 0.34 | 20.05 | 19.96 | 0.30 |
| 5.27 | 43.02 | 19.13 | 3.54 | 3.14 | 0.39 | 0.34 | 20.46 | 17.96 | 0.35 |
| 5.28 | 46.10 | 21.06 | 2.85 | 2.57 | 0.35 | 0.57 | 21.33 | 12.20 | 0.42 |
| 5.28 | 45.35 | 19.11 | 3.42 | 2.61 | 0.36 | 0.56 | 18.83 | 8.35 | 0.43 |
| 5.28 | 44.60 | 18.12 | 2.87 | 2.19 | 0.40 | 0.56 | 21.24 | 12.43 | 0.41 |
| 5.29 | 46.53 | 21.96 | 3.75 | 2.87 | 0.25 | 0.55 | 17.24 | 23.45 | 0.42 |
| 5.29 | 45.30 | 18.87 | 3.36 | 2.94 | 0.25 | 0.34 | 17.96 | 4.40 | 0.32 |
| 5.29 | 44.66 | 18.47 | 2.69 | 2.61 | 0.43 | 0.39 | 16.41 | 21.05 | 0.33 |
| 5.31 | 49.69 | 19.92 | 3.06 | 3.00 | 0.26 | 0.72 | 15.31 | 6.58 | 0.51 |
| 5.31 | ND | ND | ND | ND | ND | ND | ND | 7.85 | ND |
| 5.31 | 42.76 | 20.31 | 3.18 | 2.54 | 0.42 | 0.48 | 19.81 | 14.15 | 0.32 |
| 5.31 | 49.17 | 22.43 | 2.95 | 3.53 | 0.45 | 0.62 | 19.42 | 7.89 | 0.54 |
| 5.32 | 44.80 | 20.27 | 2.69 | 2.54 | 0.25 | 0.57 | 23.39 | 15.06 | 0.54 |
| 5.32 | 41.41 | 18.75 | 2.85 | 2.46 | 0.18 | 0.48 | 24.07 | 19.73 | 0.25 |
| 5.32 | 45.93 | 18.20 | 2.99 | 2.99 | 0.17 | 0.38 | 19.96 | 30.48 | 0.36 |
| 5.32 | 45.99 | 20.75 | 3.12 | 3.47 | 0.35 | 0.43 | 17.37 | 5.72 | 0.41 |
| 5.33 | 39.54 | 18.96 | 2.97 | 3.05 | 0.08 | 0.44 | 21.18 | 5.26 | 0.38 |
| 5.33 | 39.89 | 18.32 | 3.75 | 3.36 | 0.11 | 0.32 | 20.94 | 5.22 | 0.34 |
| 5.34 | 41.11 | 17.57 | 3.21 | 2.75 | 0.11 | 0.32 | 21.30 | ND | 0.37 |
| 5.34 | 43.06 | 17.31 | 2.72 | 1.15 | 0.40 | 0.35 | 23.60 | ND | 0.29 |
| 5.34 | 46.53 | 21.96 | 3.75 | 2.87 | 0.33 | 0.55 | 17.24 | 3.27 | 0.42 |
| 5.35 | 41.29 | 17.99 | 2.81 | 2.27 | 0.43 | 0.52 | 18.24 | 17.33 | 0.42 |
| 5.35 | ND | ND | ND | ND | ND | ND | ND | 6.94 | ND |
| 5.35 | 49.71 | 19.94 | 2.95 | 2.62 | 0.46 | 0.60 | 18.46 | 2.95 | 0.46 |
| 5.35 | 40.71 | 19.55 | 3.39 | 2.89 | 0.41 | 0.47 | 21.91 | 13.34 | 0.45 |
| 5.36 | 46.88 | 20.05 | 3.22 | 2.50 | 0.21 | 0.54 | 20.37 | 5.31 | 0.36 |
| 5.36 | 45.64 | 19.14 | 2.73 | 1.61 | 0.18 | 0.38 | 16.73 | 5.44 | 0.34 |
| 5.36 | 46.90 | 20.71 | 3.29 | 3.08 | 0.12 | 0.45 | 16.61 | 11.16 | 0.37 |
| 5.36 | 41.73 | 17.16 | 2.95 | 1.17 | 0.21 | 0.46 | 20.29 | 10.12 | 0.32 |
| 5.37 | 43.75 | 19.86 | 2.97 | 3.07 | 0.02 | 0.47 | 21.03 | 18.60 | 0.39 |
| 5.38 | 41.15 | 16.00 | 3.04 | 2.93 | 0.19 | 0.37 | 24.25 | 13.79 | 0.28 |
| 5.38 | 44.39 | 17.62 | 2.70 | 2.82 | 0.19 | 0.47 | 18.85 | 32.93 | 0.39 |
| 5.38 | 48.12 | 16.20 | 2.66 | 1.76 | 0.18 | 0.40 | 18.47 | 14.70 | 0.33 |
| 5.38 | 41.54 | 18.18 | 3.32 | 3.81 | 0.41 | 0.35 | 21.94 | 7.48 | 0.30 |
| 5.38 | ND | ND | ND | ND | ND | ND | ND | 7.76 | ND |
| 5.39 | 39.86 | 17.66 | 3.54 | 3.52 | 0.26 | 0.43 | 23.75 | 4.85 | 0.43 |
| 5.41 | 41.27 | 16.35 | 3.11 | 2.54 | 0.22 | 0.49 | 20.09 | 2.90 | 0.47 |
| 5.41 | 44.23 | 18.01 | 3.79 | 2.31 | 0.37 | 0.32 | 17.97 | 4.63 | 0.36 |
| 5.41 | 51.18 | 19.36 | 3.07 | 2.63 | 0.33 | 0.88 | 20.35 | 7.80 | 0.57 |
| 5.41 | 44.95 | 16.58 | 3.07 | 2.48 | 0.11 | 0.56 | 17.83 | 13.29 | 0.39 |
| Acetate | Glc | Xylose | Lignin (acid-soluble) | Man | Rha | Gal | Lignin (acid-insoluble) | Biomass | Ara |
| 5.41 | 50.36 | 22.24 | 2.91 | 3.42 | 0.44 | 0.35 | 18.84 | 19.64 | 0.38 |
| 5.43 | 49.52 | 15.96 | 2.94 | 2.51 | 0.37 | 0.64 | 18.11 | 12.61 | 0.36 |
| 5.43 | 45.70 | 20.14 | 3.11 | 2.86 | 0.13 | 0.52 | 17.91 | 7.62 | 0.42 |
| 5.44 | 44.17 | 19.43 | 3.09 | 3.08 | 0.30 | 0.35 | 19.41 | 13.20 | 0.31 |
| 5.46 | 38.53 | 15.94 | 3.18 | 2.49 | 0.42 | 0.44 | 18.80 | 10.75 | 0.43 |
| 5.46 | 45.67 | 18.67 | 3.37 | 3.58 | 0.19 | 0.49 | 17.58 | 13.29 | 0.46 |
| 5.47 | 41.77 | 18.21 | 2.74 | 2.00 | 0.36 | 0.37 | 19.03 |  | 0.38 |
| 5.47 | ND | ND | ND | ND | ND | ND | ND | 11.61 | ND |
| 5.47 | 42.27 | 18.92 | 3.50 | 2.73 | 0.17 | 0.46 | 23.81 | 1.54 | 0.39 |
| 5.47 | 43.02 | 19.66 | 2.77 | 2.82 | 0.42 | 0.39 | 23.54 | 7.44 | 0.37 |
| 5.48 | ND | ND | ND | ND | ND | ND | ND | 17.33 | ND |
| 5.48 | 48.81 | 21.45 | 2.86 | 2.95 | 0.19 | 0.51 | 16.03 | 13.52 | 0.45 |
| 5.49 | 47.93 | 19.75 | 2.92 | 3.12 | 0.22 | 0.53 | 19.42 | 8.80 | 0.43 |
| 5.49 | 46.35 | 18.94 | 2.98 | 2.23 | 0.19 | 0.40 | 19.18 | 13.65 | 0.37 |
| 5.49 | ND | ND | ND | ND | ND | ND | ND | 7.44 | ND |
| 5.50 | 41.33 | 18.17 | 3.06 | 2.92 | 0.38 | 0.36 | 21.48 | 13.83 | 0.34 |
| 5.51 | 42.19 | 18.39 | 2.83 | 2.88 | 0.16 | 0.50 | 21.60 | 9.57 | 0.41 |
| 5.51 | 47.40 | 18.77 | 2.60 | 2.49 | 0.22 | 0.44 | 17.55 | 28.80 | 0.35 |
| 5.52 | ND | ND | ND | ND | ND | ND | ND | 3.86 | ND |
| 5.52 | 44.82 | 15.66 | 3.22 | 2.99 | 0.29 | 0.57 | 18.41 | 2.49 | 0.41 |
| 5.52 | ND | ND | ND | ND | ND | ND | ND | 8.16 | ND |
| 5.53 | 45.95 | 19.92 | 3.20 | 3.57 | 0.40 | 0.41 | 18.07 | 5.13 | 0.45 |
| 5.54 | 46.91 | 19.65 | 3.21 | 2.63 | 0.32 | 0.63 | 15.77 | 18.60 | 0.51 |
| 5.54 | 43.48 | 17.08 | 3.17 | 2.61 | 0.20 | 0.50 | 17.50 | 3.90 | 0.40 |
| 5.55 | 43.96 | 16.51 | 2.41 | 2.77 | 0.20 | 0.42 | 19.38 | 33.52 | 0.41 |
| 5.55 | ND | ND | ND | ND | ND | ND | ND | 15.47 | ND |
| 5.56 | 42.25 | 18.92 | 2.49 | 2.68 | 0.21 | 0.42 | 18.19 | 30.39 | 0.45 |
| 5.56 | 46.26 | 21.34 | 3.53 | 3.51 | 0.41 | 0.40 | 19.20 | 11.75 | 0.35 |
| 5.57 | 47.26 | 21.37 | 3.07 | 3.21 | 0.24 | 0.61 | 20.57 | 14.06 | 0.48 |
| 5.57 | 44.37 | 20.74 | 3.04 | 3.39 | 0.42 | 0.42 | 19.25 | 6.30 | 0.40 |
| 5.57 | 47.40 | 20.80 | 3.11 | 2.92 | 0.37 | 0.51 | 16.84 | 10.61 | 0.43 |
| 5.57 | 48.28 | 18.04 | 3.43 | 2.38 | 0.45 | 0.57 | 18.01 | 18.05 | 0.40 |
| 5.58 | 43.35 | 20.49 | 2.92 | 3.01 | 0.37 | 0.50 | 18.85 | 10.34 | 0.48 |
| 5.58 | ND | ND | ND | ND | ND | ND | ND | 14.97 | ND |
| 5.58 | 42.78 | 17.45 | 3.01 | 3.00 | 0.17 | 0.46 | 21.36 | 27.49 | 0.40 |
| 5.60 | 45.91 | 20.78 | 3.54 | 3.33 | 0.30 | 0.45 | 16.26 | 11.39 | 0.39 |
| 5.61 | 49.38 | 19.25 | 3.34 | 2.39 | 0.35 | 0.61 | 15.66 | 9.66 | 0.38 |
| 5.61 | 42.62 | 18.19 | 2.81 | 2.44 | 0.17 | 0.49 | 20.81 | 5.31 | 0.39 |
| 5.61 | 47.71 | 18.04 | 2.80 | 2.80 | 0.15 | 0.44 | 17.66 | 16.19 | 0.37 |
| 5.61 | 42.38 | 23.50 | 3.66 | 4.38 | 0.30 | 0.64 | 18.30 | 10.16 | 0.43 |
| 5.63 | 40.29 | 17.75 | 3.00 | 2.48 | 0.23 | 0.52 | 23.76 | 11.70 | 0.27 |
| 5.64 | 45.40 | 19.05 | 2.90 | 2.99 | 0.42 | 0.37 | 15.77 | 12.38 | 0.30 |
| 5.64 | 40.30 | 16.47 | 2.88 | 2.96 | 0.40 | 0.61 | 22.10 | 17.60 | 0.45 |
| 5.65 | 45.32 | 19.98 | 2.99 | 3.12 | 0.36 | 0.55 | 16.98 | 15.60 | 0.44 |
| 5.65 | 44.61 | 18.15 | 2.78 | 3.17 | 0.36 | 0.51 | 18.51 | 10.89 | 0.37 |
| Acetate | Glc | Xylose | Lignin (acid-soluble) | Man | Rha | Gal | Lignin (acid-insoluble) | Biomass | Ara |
| 5.66 | 49.01 | 18.87 | 3.05 | 2.75 | 0.10 | 0.52 | 18.25 | 11.75 | 0.44 |
| 5.68 | ND | ND | ND | ND | ND | ND | ND | 13.61 | ND |
| 5.71 | 47.37 | 19.80 | 2.76 | 1.41 | 0.29 | 0.38 | 21.65 | 13.79 | 0.38 |
| 5.73 | 43.88 | 16.78 | 2.98 | 2.40 | 0.16 | 0.47 | 18.60 | 2.40 | 0.42 |
| 5.73 | 44.54 | 21.28 | 2.60 | 3.56 | 0.40 | 0.41 | 19.82 | 15.88 | 0.39 |
| 5.73 | 46.17 | 18.31 | 3.01 | 2.90 | 0.21 | 0.55 | 17.51 | 28.35 | 0.31 |
| 5.73 | 45.21 | 20.90 | 2.74 | 3.18 | 0.07 | 0.40 | 18.12 | 6.35 | 0.43 |
| 5.74 | 45.32 | 17.22 | 3.04 | 2.74 | 0.43 | 0.48 | 20.61 | 1.86 | 0.30 |
| 5.76 | 43.03 | 21.22 | 3.30 | 3.01 | 0.27 | 0.55 | 19.79 | 3.49 | 0.52 |
| 5.77 | 40.57 | 17.79 | 3.26 | 2.11 | 0.17 | 0.33 | 18.49 | 7.17 | 0.32 |
| 5.78 | 43.45 | 20.76 | 2.65 | 3.07 | 0.37 | 0.43 | 18.22 | 11.79 | 0.34 |
| 5.79 | ND | ND | ND | ND | ND | ND | ND | 23.50 | ND |
| 5.97 | 42.58 | 21.22 | 2.83 | 3.24 | 0.48 | 0.46 | 17.70 | 11.43 | 0.47 |
| 5.98 | 45.58 | 20.90 | 3.41 | 3.02 | 0.51 | 0.66 | 16.32 | 15.88 | 0.50 |
| 6.00 | 47.07 | 20.71 | 3.46 | 2.90 |  | 0.58 | 24.26 | 6.30 | 0.41 |

Glc, glucose; Man, mannose; Rha, rhamnose; Gal, galactose; Ara, arabinose.
